# Large-Scale Synthesis of Tunable Fluorescent Carbon Dots Powder for Light-Emitting Diodes and Fingerprint Identification

**DOI:** 10.3390/molecules28155917

**Published:** 2023-08-07

**Authors:** Lei Zhao, Dong Zhang, Xin Wang, Yang Li, Zihan Li, Hua Wei, Boxuan Yao, Gongtao Ding, Zifan Wang

**Affiliations:** 1 Key Laboratory of Biotechnology and Bioengineering of State Ethnic Affairs Commission, Biomedical Research Center, Northwest Minzu University, Lanzhou 730030, China; 2 School of Chemical Engineering, Northwest Minzu University, Lanzhou 730030, China; d874724068@163.com; 3 School of Chemistry and Chemical Engineering, Qilu University of Technology (Shandong Academy of Sciences), Jinan 250353, China; wx19971221@163.com (X.W.); zh17860658513@163.com (Z.L.); wh17861406068@163.com (H.W.); ybx20021218@163.com (B.Y.); 4 Lanzhou Hualian Xinminao Dental Clinic, Lanzhou 730000, China; lwr03067719@s.ytu.edu.cn

**Keywords:** tunable carbon dots, large-scale synthesis, solid-phase synthesis, light-emitting diodes, fingerprint identification

## Abstract

The emergence and fast development of carbon dots (CDs) provide an unprecedented opportunity for applications in the field of photoelectricity, but their practicability still suffers from complicated synthesis procedures and the substrate dependence of solid-state fluorescence. In this study, we design a unique microwave-assisted solid-phase synthesis route for preparing tunable fluorescent CD powders with yellow, orange, and red fluorescence (Y-CDs, O-CDs, R-CDs) by simply adjusting the mass ratio of reactants, a method which is suitable for the large-scale synthesis of CDs. The Y-/O-/R-CDs were systematically characterized using physics and spectroscopy techniques. Based on the perfect solid-state fluorescence performance of the proposed fluorescent CD powders, the Y-/O-/R-CDs were successfully applied for the construction of multi-color and white light-emitting diode devices at low cost. Furthermore, the Y-CDs displayed much higher yield and luminous efficiency than the O-CDs and R-CDs and were further used for fingerprint identification on the surfaces of glass sheets and tinfoil. In addition, the R-CD aqueous solution fluorescence is sensitive to pH, suggesting its use as a pH indicator for monitoring intracellular pH fluctuations. The proposed series of fluorescent powders composed of CDs may herald a new era in the application of optical components and criminal investigation fields.

## 1. Introduction

**Scheme 1:**
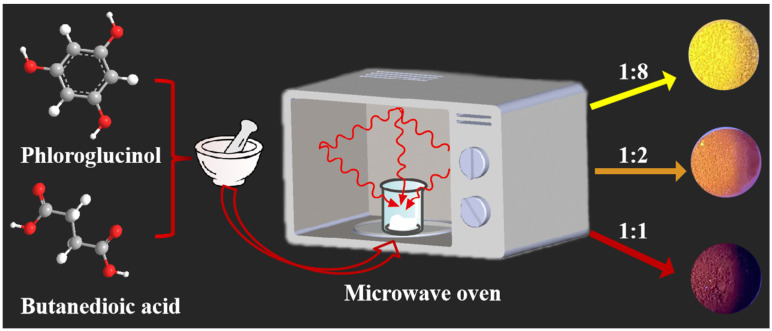
The synthesis of fluorescent CD powder and its application in light-emitting diodes and fingerprint identification.

The rapid development of fluorescent nanomaterials, including semiconductor quantum dots [1], metal nanoclusters [2], rare earth nanoparticles [3], and perovskite quantum dots [4], not only provides an unprecedented opportunity for analytical chemistry and bioimaging, but it also establishes a robust foundation for the production of optical elements and trace inspection [5,6,7]. However, the further practical applications of the above-mentioned fluorescent nanomaterials are still limited by their costly precursors, complicated synthesis procedures, or high toxicity, which could lead to high environmental and biological risks [8,9,10]. Therefore, it is imperative to develop inexpensive and harmless new fluorescent nanomaterials. Carbon dots (CDs), a new type of fluorescent carbon-based nanomaterial proposed first in 2004, have attracted significant attention due to their advantages of being environmental-friendly and having good biocompatibility, unique optical properties, stability, and tunability, and low cost, resulting in them being considered as an effective alternative to heavy metal-based semiconductor quantum dots [11,12,13]. In recent years, CDs have been widely applied in various fields, such as chemical sensing [14], biosensing [15], biomedical applications [16], and photocatalysis [17]. They have also shown great potential in the light-emitting devices and fingerprint identification fields [18,19,20]. The synthesis of CDs usually includes top-down and bottom-up methods, whereby top-down approaches obtain CDs by cracking large carbon nanomaterials, such as graphene [21]. Conversely, the bottom-up processes provide broad opportunities to obtain CDs of various sizes, shapes, and properties through pyrolyzing or carbonizing small organic or aromatic molecules. As typical representatives of bottom-up methods, solvothermal and microwave methods have been widely applied for the synthesis, performance improvement, and fluorescent emission adjustment of CDs in recent years, and these methods have significantly promoted the development of CDs for practical application [22,23].The fluorescence efficiency of CD solutions has been improved after more than ten years of research, which is reflected in improvements in the quantum yield and tunability of emission wavelengths. Most CDs can emit apparent fluorescence and keep their photoluminescence properties in various organic solvents with different polarities, such as ethanol, dimethyl sulfoxide, acetone, etc. Notably, although most reported CD-based fluorescent materials possess excellent optical properties in solvents, the solid-state fluorescence is hard to maintain due to marked aggregation-induced quenching effects [24,25,26]. In the light-emitting devices field, it commonly necessary to introduce different substrates, such as BaSO_4_, starch, and SiO_2_, to retain the solid-state fluorescence of CDs, which is inconvenient for practical application [27,28,29]. Zhang et al. developed three-emission color CDs with good water dispersibility and strong emission using the green hydrothermal method. However, the self-quenching effect in the solid state was difficult to avoid unless a polyvinyl alcohol matrix was introduced to yield solid-state films [30]. Therefore, developing high-efficiency CD-based solid fluorescent material without any dispersive medium is urgent. Up to now, some researchers have focused on synthesizing and applying light-emitting diodes and fingerprint imaging of CD-based solid fluorescent material [31,32,33]. For example, Wang et al. reported a series of solid-state fluorescent CDs using phloroglucinol and urea as sources via the one-step microwave method by regulating the reactant ratio and microwave power [34]. These CDs showed a good self-quenching resistance effect and were successfully applied for fabricating light-emitting diodes, but the synthesis route still suffers from solvent dependence. In contrast, Xiong et al. obtained well-fluorescing CD powder via a simple solid-state reaction within 3 h, which was further applied for fingerprint identification. However, the proposed CDs preparation process was still time-consuming [35]. In addition, the large-scale synthesis of CD-based solid fluorescent material commonly requires high temperature/pressure and high energy consumption, which limit this method’s development into an industrial process. Hence, the solvent-free, rapid, low-cost, large-scale synthesis of fluorescent CD powders is still challenging. Inspired by the great application potential of CD-based solid-state fluorescent material in light-emitting devices combined with the bottleneck problems of its synthesis methodology, in this work, we propose an effective microwave-assisted solid-phase reaction strategy for synthesizing high-performance fluorescent CD powder (Scheme 1). CDs with perfect solid-state fluorescence can be obtained by simply pyrolyzing a phloroglucinol and succinic acid mixture within 2 min with no extra solvent participation. Yellow, orange, and red fluorescent CD powders (Y-CDs, O-CDs, R-CDs) can be synthesized easily by adjusting the ratio of reactants, producing high yields of 61%, 43%, and 32%, respectively, which are suitable for large-scale preparation and low-cost application. Based on their excellent solid-state fluorescence properties, the prepared fluorescent CDs powders were successfully used for the construction of multi-color light-emitting diodes (LEDs) with good color rendering index. Furthermore, the proposed CDs also exhibit excellent application potential in fingerprint identification. Additionally, the fluorescence signal of R-CDs in aqueous solution is sensitive to pH change, which can be used as a pH indicator for monitoring intracellular pH fluctuations. This study may provide a valuable reference for the simple synthesis and practical commercial application of CD-based solid-state fluorescent materials.

## 2. Results and Discussion

### 2.1. Synthesis of Y-CDs, O-CDs and R-CDs

**Figure 1:**
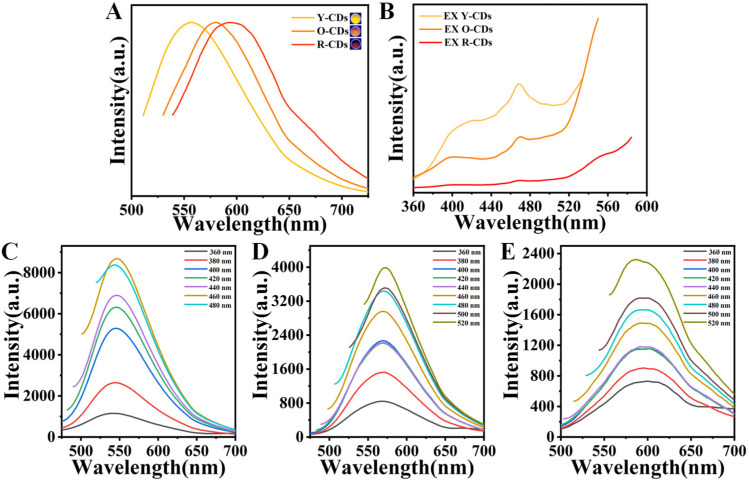
The normalized emission (**A**) and excitation spectra (**B**) of Y-CDs, O-CDs, and R-CDs; the emission spectra of Y-CDs (**C**), O-CDs (**D**), and R-CDs (**E**) under different excitation wavelengths.

To obtain a series of high-efficiency CD-based fluorescent powders with different emission wavelengths, we propose a particular solid-phase synthesis strategy for preparing tunable fluorescent CD powders. This synthesis strategy utilizes phloroglucinol and butanedioic acid as raw materials and simple adjustments to their mass ratio. The solid-state fluorescence of the proposed CDs should be unique to the large conjugate structure formed from the reaction of phloroglucinol and butanedioic acid. The fluorescence emission wavelength of the CDs is associated with the reactants’ ratio, and the emission wavelength of the CDs red-shift is due to the formation of larger conjugate structures with increasing ratios of phloroglucinol and butanedioic acid. As shown in Figure 1A, the Y-CDs with a light center at about 550 nm can be obtained easily with the mass ratio of phloroglucinol and butanedioic acid of 1:8. When adjusting the mass ratio to 1:2, we can prepare O-CDs with prominent orange fluorescence at an emission wavelength of 580 nm. Furthermore, the R-CDs with an emission wavelength of 600 nm can also be synthesized successfully under the mass ratio of 1:1. Similarly, the band-gaps of Y-CDs, O-CDs and R-CDs are 2.34 eV, 2.16 eV, and 2.02 eV, respectively, which were calculated from UV–Vis spectra. In addition, the yields of Y-CDs, O-CDs, and R-CDs can reach 61%, 43%, and 32%, respectively, while their lifetimes are 5.2 ns, 7.8 ns, and 7.3 ns, respectively. From Figures S1 and S2, we can conclude that phloroglucinol or butanedioic acid alone cannot be used for synthesizing fluorescent CD powder directly. The solid-state fluorescence of the proposed CDs is dependent on the synergistic reaction of phloroglucinol and butanedioic acid. Furthermore, the proposed strategy still possesses the advantages of being solvent-free and having a short reaction time, which are characteristics suitable for the large-scale, emerging synthesis of tunable CD-based fluorescent powders. From the excitation spectra, we can see that the fluorescence of the as-prepared fluorescent powders has a broad wave band characteristic (Figure 1B). In addition, as shown in Figure 1C–E, the emission centers of Y-/O-/R-CDs were independent of excitation wavelengths, which suggests that the developed CD-based fluorescent powders have uniform luminescence centers [36]. Hence, the simple synthesis route, high yield, and consistent luminescence properties of the proposed CDs lay a solid foundation for further optical applications. The fluorescence spectra of the Y-/O-/R-CDs water solutions also were studied. As shown in Figure S3, the Y-CDs and O-CDs could not emit evident fluorescence in the aqueous phase, whereas the R-CDs aqueous solution emitted bright green fluorescence with an emission center at 535 nm. The excellent solid-state fluorescence of Y-CDs may originate from an aggregation-induced fluorescence enhancement effect, and the relatively weak solid-state fluorescence of R-CDs could be attributed to their aggregation-induced fluorescence quenching effect. The good fluorescence performance of the R-CDs aqueous solution inspired us to research it systemically. Figure S4 shows that the R-CDs aqueous solution displayed a broad excitation band with an optimal excitation wavelength at 470 nm. Furthermore, the fluorescence emission wavelengths of R-CDs aqueous solution depended on changes in the excitation wavelength. With the excitation wavelength changing from 300 nm to 500 nm at an interval of 20 nm, the emission peaks red-shift gradually, as shown in Figure S5. The UV–Visible absorption spectrum of the CDs aqueous solution is shown in Figure S6. Three prominent absorption peaks at 275 nm, 315 nm, and 474 nm can be observed easily. The 275 nm and 315 nm peaks can be assigned to the n-π* transition. The broad absorption band at about 474 nm could be assigned to the coupling of the π-state, which also matches the optimal excitation wavelength. All the above results indicate that R-CDs have similar optical properties to typical CDs. The R-CDs were synthesized using phloroglucinol and butanedioic acid as raw materials, so we speculate that the fluorescence signal of the R-CD aqueous solution may be sensitive to pH change due to the protonation and deprotonation effect of hydroxyl and carboxyl functional groups from the surface of the R-CDs. As shown in Figure S7, the R-CDs aqueous solution can emit weak fluorescence at pH < 5.20, and the fluorescence intensities increase sharply when the pH increases from 5.60 to 7.62. When the pH further increases to 8.52, the fluorescence change in the R-CDs aqueous solution can be ignored. Figure S8 shows that the fluorescence intensity changes linearly with pH, with a correlation coefficient of 0.9988 in the range of 5.60 to 7.62. The fluorescence intensity of the R-CD aqueous solution at pH 7.62 was almost seven-fold higher than that at pH 5.60, which proves the high sensitivity of the fluorescence signal of the R-CD aqueous solution to pH change. Owing to the high sensitivity of the R-CD water-phase fluorescence to pH change, the R-CDs can be used as an effective pH indicator for monitoring intracellular pH fluctuations. As shown in Figure S9, no obvious fluorescence signal could be captured from the unstained cells, but the fluorescence signal of the cells showed a noticeable difference with pH at 6.0 and 7.4 after staining by R-CDs, which indicates that the R-CDs can enter the living cells and distinguish the pH fluctuations in cells.

### 2.2. Characterization of Y-CDs, O-CDs and R-CDs

**Figure 2:**
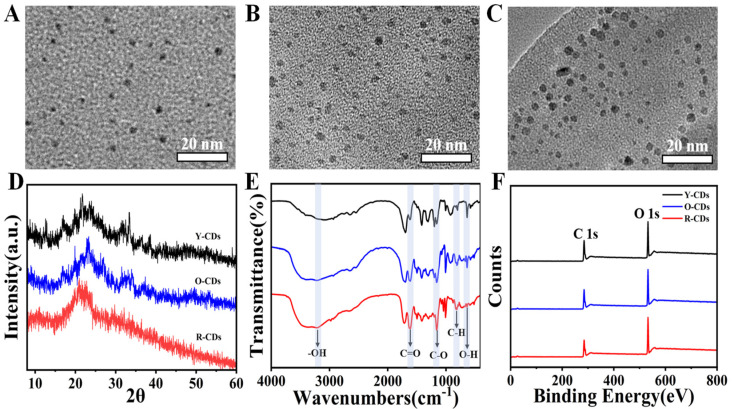
The TEM images of Y-CDs (**A**), O-CDs (**B**), and R-CDs (**C**) fluorescent powders; the full XRD (**D**), FTIR (**E**), and XPS (**F**) spectra of Y-CDs, O-CDs and R-CDs fluorescent powders.

**Figure 3:**
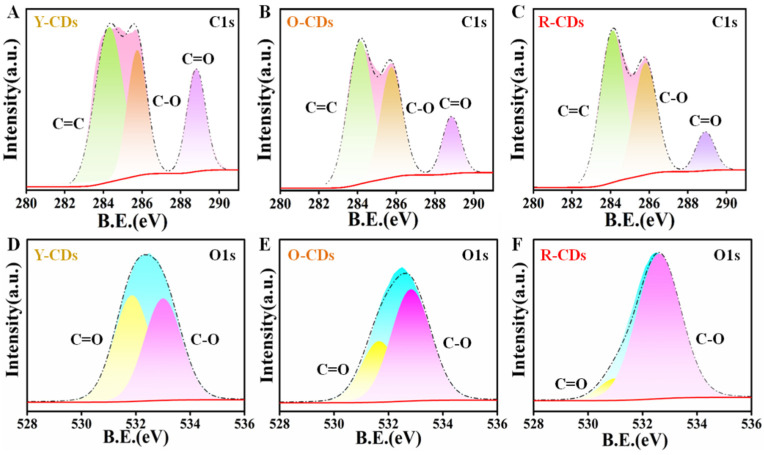
The high-resolution C1s (**A**–**C**) and O1s (**D**–**F**) spectra (**B**) of synthesized Y-CDs (**A**,**D**), O-CDs (**B**,**E**), and R-CDs (**C**,**F**).

To ensure their suitability for application, the morphology and size of the synthesized Y-/O-/R-CDs were studied by TEM. As shown in Figure 2A–C, all the developed CD-based fluorescent powders show good monodispersity, with nearly spherical nanoparticles and a size range from 2 to 4 nm (Figure S10). The XRD patterns indicate the synthesized fluorescent CD powders are amorphous (Figure 2D). Furthermore, the surface property of the proposed CDs were characterized by the FTIR spectrum. Figure 2E shows that the as-prepared Y-/O-/R-CDs powders are similar in composition, and the characteristic absorption peak of -OH in phloroglucinol can be observed at 3293.2 cm^−1^. Furthermore, we can observe the stretching vibration of C=O and C-O at 1714.4 cm^−1^ and 1147.6 cm^−1^, respectively, on the surface of the CD-based fluorescent powders [37]. The out-of-plane bending vibration peaks of C-H and O-H can be found at 820.3 cm^−1^ and 680.1 cm^−1^, respectively. Especially with an increasing mass ratio of phloroglucinol and butanedioic acid, the content of the carbonyl functional group decreased gradually from Y-CDs to R-CDs. These results indicate that the surfaces of the prepared Y-/O-/R-CDs were rich in oxygen-containing functional groups. Further information on the chemical composition and structure of Y-/O-/R-CDs was obtained from the XPS. As shown in the full XPS spectra of Y-/O-/R-CDs (Figure 2F), two typical peaks at 284.5 eV and 532.5 eV can be observed quickly, corresponding to C 1s and O 1s, respectively. The proposed series of CDs contains only the elements carbon and oxygen, resulting in lower environmental risks due to elimination of the overly dependent nitrogen element in the synthesis of CDs [38]. In Figure 3A–C, the C=C (284.1 eV), C-O (285.8 eV), and C=O (288.8 eV) bands in the Y-/O-/R-CDs were observed clearly from the high-resolution C 1s spectrum [39]. The O 1s displays two typical peaks at 531.0 eV and 532.6 eV, which can be attributed to the C=O and C-O bands, respectively, of the Y-/O-/R-CDs [40]. From Table S1, we can further confirm that the percentage of the carbonyl functional group decreased along the gradient from Y-CDs to O-CDs to R-CDs. These results are all consistent with the FITR data analysis. The results of XPS indicate that the proposed Y-/O-/R-CDs without nitrogen possess low environmental risk, and consequently, potential eutrophication effects can be avoided in practical applications.

### 2.3. The Biocompatibility of Y-CDs, O-CDs, R-CDs

**Figure 4:**
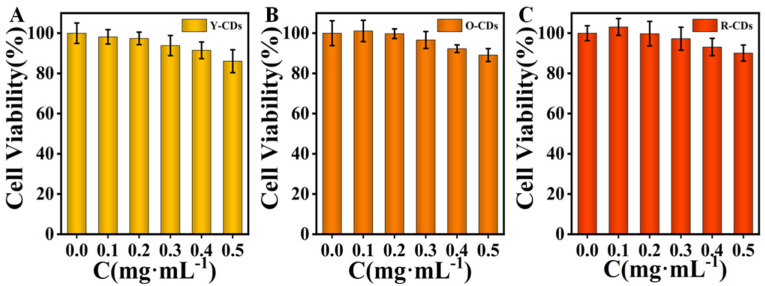
MTT experiment results for Y-CDs (**A**), O-CDs (**B**), and R-CDs (**C**).

To assess their suitability for application, MTT assays were carried out to evaluate the cytotoxicity of Y-/O-/R-CDs on Hela cells. As shown in Figure 4, the as-prepared Y-/O-/R-CDs fluorescent powders exhibited good biocompatibility, and the cell viability results were all higher than 85% when the concentration of Y-/O-/R-CDs was set as 500 μg·mL^−1^. In addition, the synthesized Y-/O-/R-CDs also possess good solid-state fluorescence stability, as shown in Figure S11. The fluorescence performance of Y-/O-/R-CDs was retained at 90% after storing for 60 days, further demonstrating the utility of Y-/O-/R-CDs in practical applications.

### 2.4. Application of Y-CDs, O-CDs, and R-CDs in Multi-Color/White LEDs

**Figure 5:**
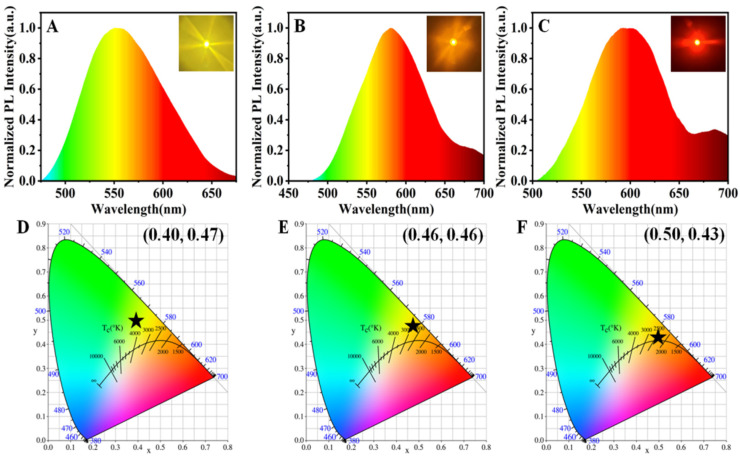
Emission spectra (**A**–**C**) and CIE coordinates (**D**–**F**) of yellow, orange and red LEDs (inset: photographs of yellow, orange and red LEDs).

**Figure 6:**
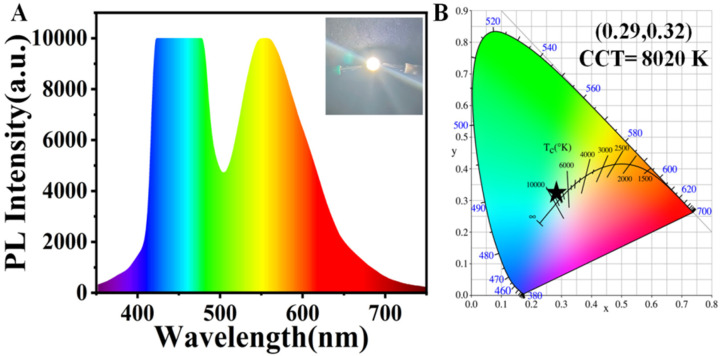
Emission spectrum (**A**) and CIE coordinates (**B**) of white LED (inset: photograph of white LED).

LEDs have become popular lighting devices because of several advantages, including their adjustable spectrum, long life, and high efficiency [41,42]. Based on the excellent solid-state fluorescence properties of the prepared series of CDs, a variety of LEDs can be fabricated using CDs as the luminescence conversion layer, including multi-color LEDs and white LEDs. Firstly, the CDs with three fluorescent colors were used as phosphors and combined with UV chips to produce multi-color LEDs. Figure 5 shows that the Commission Internationale de L’Eclairage (CIE) coordinates of the yellow, orange, and red LEDs are located at (0.40, 0.47), (0.46, 0.46) and (0.50, 0.43), respectively, and can emit bright yellow, orange and red light. Furthermore, a cold-white LED (Figure 6A inset) with a correlated color temperature (CCT) of 8020 K and CIE coordinates of (0.29, 0.32) (Figure 6B) was prepared by using a yellow solid-state fluorescent powder as the phosphor and combining it with a 450 nm blue chip. The cold-white LED had a high color reduction degree, which is more suitable for exhibition halls and other environments [43]. Therefore, the designed tunable CDs may have appropriate photoelectric element field applications.

### 2.5. Fingerprint Identification Application

**Figure 7:**
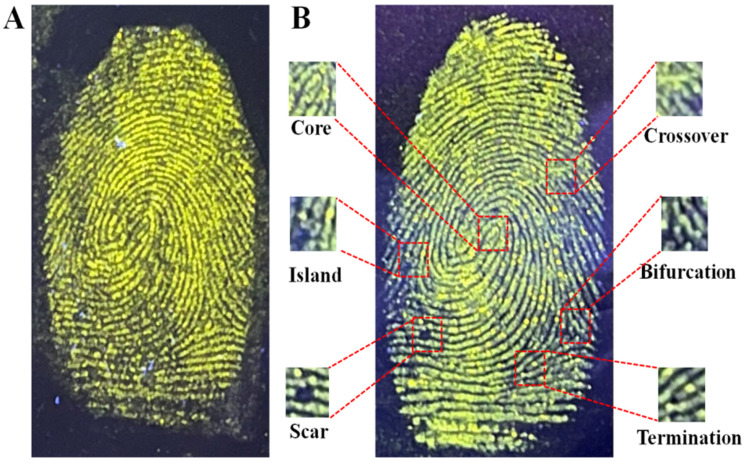
Fluorescence images of latent fingerprints on glass sheet (**A**) and tinfoil (**B**) using Y-CD powder with UV light irradiation.

Moreover, the CDs with excellent properties are promising for fingerprint identification. Firstly, CDs are almost non-toxic, which is very beneficial for the operator; secondly, the strong fluorescence signal of CDs can significantly enhance contrast, improve resolution and reduce background interference. Compared with O-CDs and R-CDs, the synthesized Y-CDs exhibited stronger solid-state fluorescence and higher yield, which was effectively employed as a phosphor powder utilizing the dusting method for imaging fingerprints on glass sheets and tinfoil [42]. As shown in Figure 7, the fingerprint images with bright yellow fluorescence on glass sheets and tin foil could be distinguished clearly under UV light irradiation (365 nm). All details of the fingerprint image on the surface of the tinfoil can be identified, including the core, islands, scar, crossover, bifurcation, and termination (Figure 7B). The details of these fingerprint images were consistent on different substrates, confirming that the dusting method with the proposed Y-CDs is reproducible and reliable. Therefore, the developed Y-CDs may have potential applications in criminal investigation.

## 3. Materials and Methods

### 3.1. Materials

Phloroglucinol and butanedioic acid were purchased from Aladdin Biological Technology Co., Ltd., (Shanghai, China). 3-(4,5-dimethyl-2-thiazolyl)-2,5-diphenyl-2-H-tetrazolium bromide (MTT) agent was obtained from Shanghai Macklin Biochemical Co., Ltd., (Shanghai, China). Shenzhen Looking Long Technology Co., Ltd., (Shenzhen, China) supplied the organic silicone and epoxy resins. All other reagents were of analytical grade and used without further purification. Ultrapure water with a resistivity of 18.25 MΩ·cm was used throughout the experiment (UPR-II-10T, Sichuan Youpu Ultra Pure Technology Co., Ltd., Sichuan, China).

### 3.2. Characterization

The photoluminescence (PL) spectra of the synthesized Y-CDs, O-CDs, and R-CDs were obtained using an F97Pro fluorescent spectrophotometer (Shanghai Lengguang Technology Co., Ltd., Shanghai, China). A NEXUS 670 Fourier was used to measure the elemental composition, and the molecular structure of Y-CDs, O-CDs, and R-CDs was examined using transform infrared spectroscopy (FTIR, Nicolet, Thermo Fisher Scientific, Columbia, MD, USA), and an EscaLab Xi+ X-ray photoelectron spectroscopy (XPS, Thermo Fisher Scientific, Columbia, MD, USA). The morphology and size of the prepared Y-CDs, O-CDs, and R-CDs were characterized by transmission electron microscopy (TEM, FEI Tecnai G2 F30, Thermo Fisher Scientific, Columbia, MD, USA) with a 200 kV accelerating voltage and dynamic light scattering (BI-200SM, Brookhaven, Bruker Corporation, Karlsruhe, Germany). The MTT test was performed on an RT-6100 enzyme-mark analyzer (Rayto, Shenzhen, China) to assess the biological risk of the obtained CDs.

### 3.3. Preparation of Y-CDs Fluorescent Powder

In a typical process, 0.05 g of phloroglucinol and 0.4 g of butanedioic acid were placed in an agate mortar and ground into a uniform powder. Then, the mixture was transferred into a 50 mL beaker. After heating for 5 min using a domestic microwave oven (700 W), the powder changed from white to yellow, indicating Y-CDs formation.

### 3.4. Preparation of O-CDs Fluorescent Powder

Similarly, 0.15 g of phloroglucinol and 0.30 g of butanedioic acid were placed in an agate mortar and ground into a uniform powder. Then, the mixture was transferred into a 50 mL beaker. After heating for 5 min using a domestic microwave oven (700 W), the powder changed from white to orange, indicating the production of O-CDs.

### 3.5. Preparation of R-CDs Fluorescent Powder

In a typical process, 0.23 g of phloroglucinol and 0.23 g of butanedioic acid were placed in an agate mortar and ground into a uniform powder. Then, the mixture was transferred into a 50 mL beaker. After heating for 5 min using a domestic microwave oven (700 W), the powder changed from white to brownish black, indicating the formation of R-CDs.

### 3.6. MTT Assay

A standard MTT assay was carried out to evaluate the cell cytotoxicity of the developed CDs. Hela cells were seeded in a 96-well plate and allowed to adhere overnight. The culture medium was removed after incubation for 24 h at 37 °C, and DMEM medium containing the CDs was used to treat the Hela cells at different concentrations (0, 100, 200, 300, 400, and 500 μg·mL^−1^) for another 24 h. Then, the incubation solution was removed and a 10% MTT agent culture medium was added. After incubation for another 4 h, the supernatant was discarded, and 150 µL of dimethyl sulfoxide was added to dissolve the formazan crystals. The absorbance values at 490 nm were measured using a microplate reader (Multiskan FC Type 357).

### 3.7. The Fabrication of LEDs Devices

LED chips with central emission values of 365 nm and 450 nm were purchased from Yuanyue photoelectric technology Co., Ltd., (Shenzhen, China) Using the solid fluorescent powder of Y-CDs, O-CDs, and R-CDs as phosphors, the working voltage was set at 3 V, and the 365 nm UV chip was used as a base. The fluorescent CD powder was dissolved in an epoxy hardener. The obtained solution was mixed with a hardener at a 4:1 volume ratio. After curing the mixture for 1 h at 100 °C, yellow, orange, and red LEDs were successfully fabricated. A white LED device was fabricated using the solid fluorescent powder of Y-CDs as phosphors. The working voltage was set at 3 V, and the 450 nm blue light chip was used as a base. The other steps are similar to those described above.

## 4. Conclusions

This study proposes an ultra-fast microwave-assisted solid-phase synthesis method for preparing tunable fluorescent CD powders without involving extra solvent. The adjustment of the fluorescence of the CD powder from yellow to orange and then to red was realized by simply controlling the mass ratio of reactants. With their high-yield, excellent optical properties, and low cytotoxicity, the developed Y-/O-/R-CDs were successfully applied in fabricating low-cost multi-color LEDs. Furthermore, the Y-CDs with higher luminous efficiency and yield were further used for the construction of cold-white LEDs and for multi-matrix fingerprint identification with adequate sensitivity. Therefore, Y-CDs may be an effective candidate material for lighting and criminal investigation applications. In addition, the R-CDs aqueous solution fluorescence was sensitive to pH change, which can be used as an effective pH sensor for monitoring intracellular pH fluctuations. This study opens new horizons for simple and large-scale synthesis of CDs and promotes the practical development from the laboratory to commercialization of CD-based fluorescent materials.

## Data Availability

The dataset generated and analyzed in this study is not publicly available but may be obtained from the corresponding author upon reasonable request.

## Supplementary Materials

The following supporting information can be downloaded at: https://www.mdpi.com/article/10.3390/molecules28155917/s1. Figure S1: The emission spectrum of obtained product using only phloroglucinol as a reactant; Figure S2: The emission spectrum of obtained product using only butanedioic acid as a reactant; Figure S3: The emission spectra of Y-/O-/R-CDs aqueous solutions; Figure S4: The excitation spectrum of R-CDs aqueous solution; Figure S5: The emission spectra of R-CDs aqueous solution under various excitation wavelengths; Figure S6: The UV–Visible spectrum of R-CDs aqueous solution; Figure S7: The emission spectra of R-CDs aqueous solutions at different pH levels; Figure S8: The linear relation between the fluorescence intensity of R-CDs aqueous solutions and pH change; Figure S9: The fluorescent microscopy images of unstained Hela cells and staining by R-CDs under pH 6.0 and 7.0; Figure S10: The fluorescence intensities of Y-/O-/R-CDs fluorescent powder after storing for different numbers of days; Figure S11: The fluorescence intensities of Y-/O-/R-CDs fluorescent powder after storing different days; Table S1: Values (%) of each peak fitted in O1s.

## Sample Availability

Samples of the compounds are available from the authors.
