# Supplementary material for: Large-Scale Synthesis of Tunable Fluorescent Carbon Dots Powder for Light-Emitting Diodes and Fingerprint Identification
Source: Molecules. 2023 Aug 7;28(15):5917. doi: 10.3390/molecules28155917 (PMC10421340; doi:10.3390/molecules28155917)
Supplement: Supplementary file 1 [file molecules-28-05917-s001.zip › molecules-2447357-supplementary.pdf]

Large-scale synthesis of tunable fluorescent carbon dots powder for light-emitting diodes and fingerprint identification

Lei Zhao[1,*], Dong Zhang[3], Xin Wang[2], Yang Li[4], Zihan Li[2], Hua Wei[2], Boxuan Yao[2], Dinggong Tao[1,*], Zifan Wang[1,*]

[1]*Key Laboratory of Biotechnology and Bioengineering of State Ethnic Affairs Commission, Biomedical Research Center; Engineering Research Center of Key Technology and Industrialization of Cell-based Vaccine, Ministry of Education; Gausu Tech Innovation Center of Animal; China-Malaysia National Joint Laboratory, Northwest Minzu University, Lanzhou, 730030, P. R. China.*

[2]*School of Chemistry and Chemical Engineering, Qilu University of Technology (Shandong Academy of Sciences), Jinan, 250353, P. R.China.*

[3] *School of Chemical Engineering, Northwest Minzu University, Lanzhou, 730030, P. R. China.*

[4] *Lanzhou Hualian Xinminao Dental Clinic, Lanzhou, 730000, P. R. China.*

* Corresponding author.

E-mail: zhaoleixbmz@163.com (L. Zhao), dinggongtao@outlook.com (G. Ding),

zifan.wang0316@qq.com (Z. Wang)

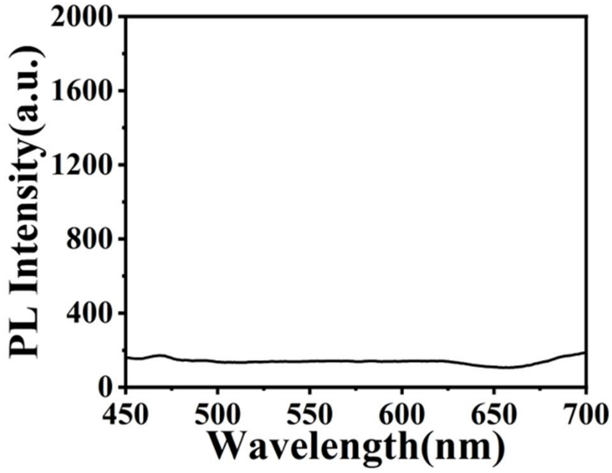

Figure S1. The emission spectrum of obtained product just using phloroglucinol as reactant.

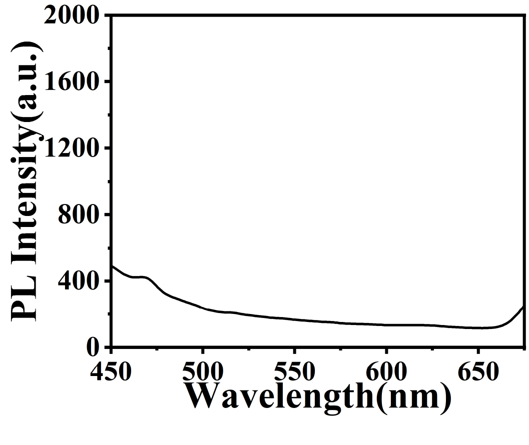

Figure S2. The emission spectrum of obtained product just using butanedioic acid as reactant.

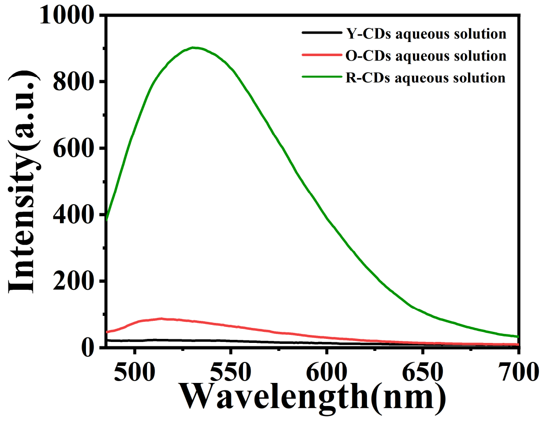

Figure S3. The emission spectra of Y-/O-/R-CDs aqueous solution.

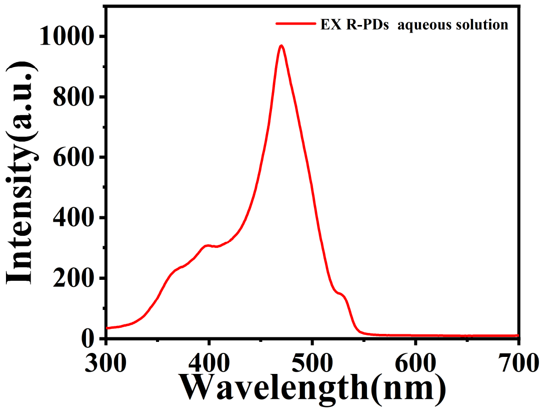

Figure S4. The excitation spectrum of R-CDs aqueous solution.

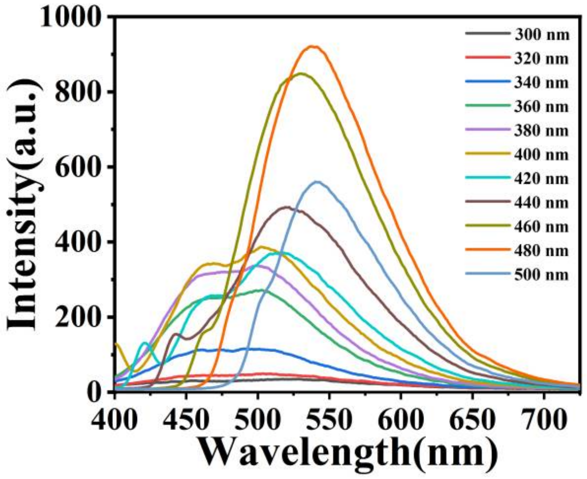

Figure S5. The emission spectra of R-CDs aqueous solution under excitation wavelengths.

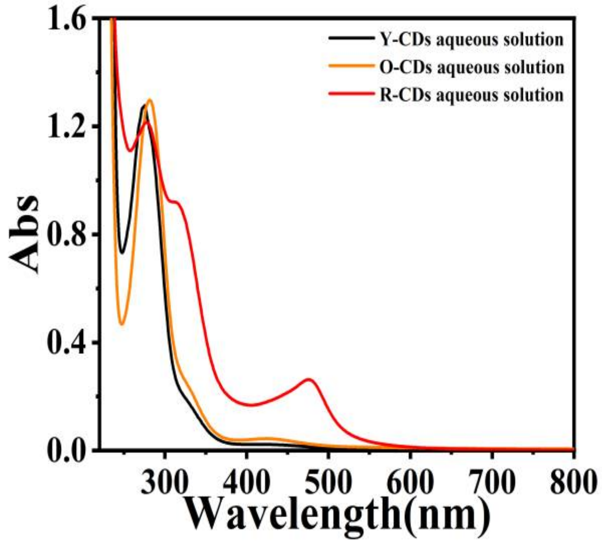

Figure S6. The UV-Visible spectra of Y-/O-/R-CDs aqueous solution.

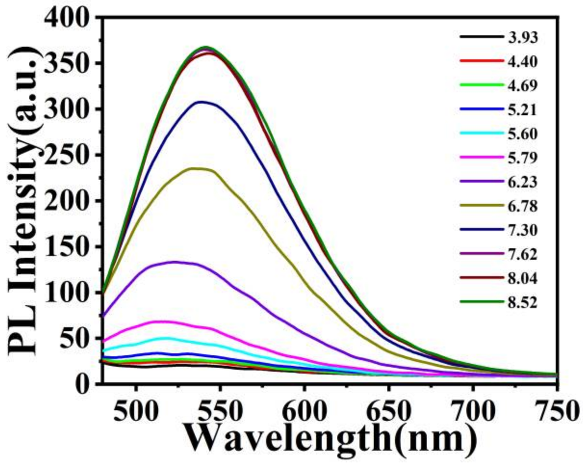

Figure S7. The emission spectra of R-CDs aqueous solution with different pH.

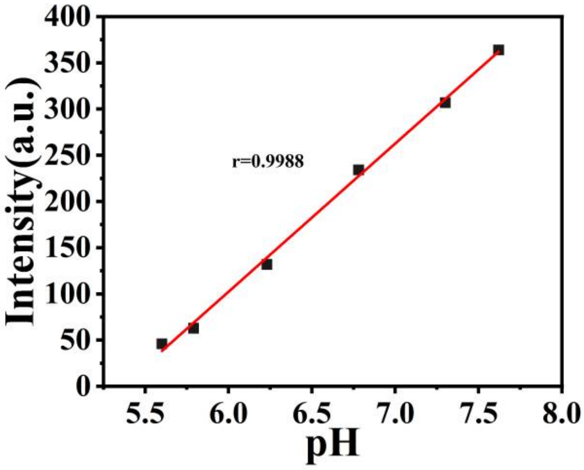

Figure S8. The linear relation between the fluorescence intensity of R-CDs aqueous solution and pH change.

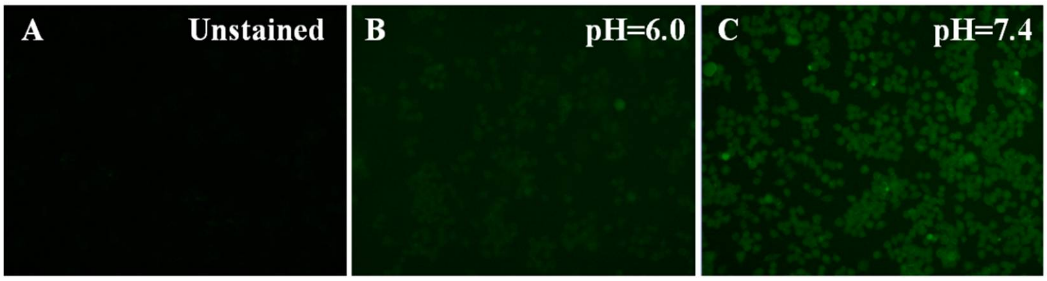

Figure S9. The fluorescent microscopy images of unstained Hela cells and staining by R-CDs under pH 6.0 and 7.0.

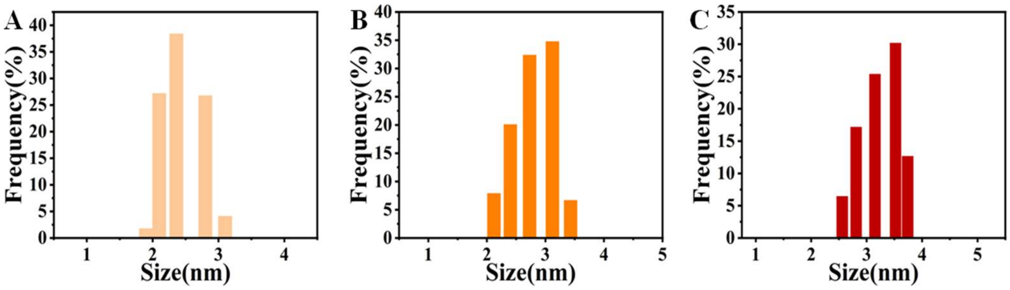

Figure S10. The size distribution of Y-CDs (A), O-CDs (B), and R-CDs (C).

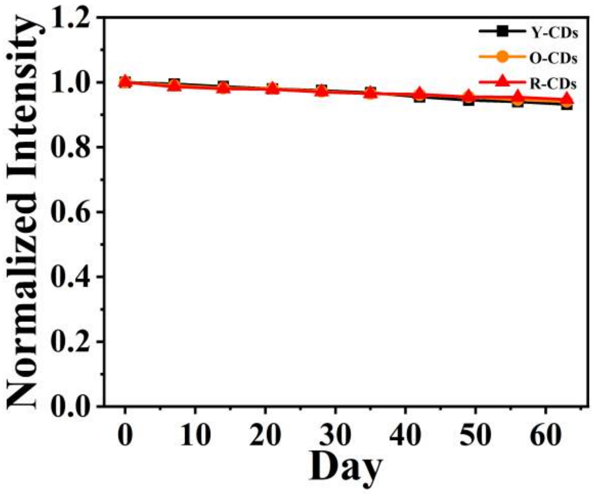

Figure S11. The fluorescence intensities of Y-/O-/R-CDs fluorescent powder after storing different days.

Table S1 Values (%) of each peak fitted in O1s.

	C=O(%)	C-O(%)
Y-CDs	51.2	48.8
O-CDs	30.6	69.4
R-CDs	10.6	89.4
